# Development of a pediatric ophthalmology academic partnership between Canada and Ethiopia: a situational analysis

**DOI:** 10.1186/s12909-020-02368-y

**Published:** 2020-11-16

**Authors:** Stephanie N. Kletke, Jibat G. Soboka, Helen Dimaras, Sadik T. Sherief, Asim Ali

**Affiliations:** 1 Department of Ophthalmology and Vision Sciences, University of Toronto, Toronto, Canada; 2 Department of Ophthalmology, School of Medicine, College of Health Sciences, Addis Ababa University, P.O. Box 9086, Addis Ababa, Ethiopia; 3 Department of Ophthalmology, Menelik II Hospital, Addis Ababa, Ethiopia; 4 Department of Ophthalmology and Vision Sciences, The Hospital for Sick Children, Toronto, Canada; 5 Division of Clinical Public Health, Dalla Lana School of Public Health, University of Toronto, Toronto, Canada; 6 Child Health Evaluative Sciences Program, SickKids Research Institute, Toronto, Canada; 7 Department of Human Pathology, College of Health Sciences, University of Nairobi, Nairobi, Kenya

**Keywords:** Pediatric ophthalmology fellowship, Academic partnership, Ethiopia, Toronto Addis Ababa academic collaboration, Situational analysis, Needs assessment

## Abstract

**Background:**

Educational capacity building in pediatric ophthalmology is necessary to address the burden of childhood blindness in Ethiopia. Residency and fellowship training at Addis Ababa University (AAU) have been enhanced with support from the University of Toronto (UofT), following the established Toronto Addis Ababa Academic Collaboration (TAAAC). Our aim was to assess the feasibility of implementing a pediatric ophthalmology fellowship at AAU with support from UofT, modeled by successful postgraduate medical education within TAAAC.

**Methods:**

A situational analysis, including a needs assessment, was conducted at Menelik II Hospital, Addis Ababa. Staff expertise, equipment and infrastructure were compared to International Council of Ophthalmology fellowship guidelines. Patient volumes were assessed through medical chart review. Local training needs were evaluated. A strategic working meeting facilitated program specification.

**Results:**

The faculty consisted of 11 ophthalmologists, including 2 pediatric specialists. Fourteen thousand six hundred twenty-seven medical and three thousand six hundred forty-one surgical pediatric cases were seen in the previous year. A 2-year fellowship incorporating anterior segment, retinoblastoma, strabismus, and retinopathy of prematurity modules was developed. Research collaborations, didactic teaching, and surgical supervision were identified as priorities requiring support. Quality standard indicators included faculty feedback, case log review and formal examination. Telemedicine, development of a larger eye hospital and partnerships to support equipment maintenance were identified as strategies to manage implementation barriers.

**Conclusions:**

The situational analysis provided a way forward for the development of a pediatric ophthalmology fellowship, the first of its kind in Eastern Africa. Learning outcomes are feasible given high patient volumes, qualified staff supervision and sufficient equipment. Strategic partnerships may ensure resource sustainability.

**Supplementary Information:**

The online version contains supplementary material available at 10.1186/s12909-020-02368-y.

## Background

Globally, an estimated 19 million children suffer from visual impairment [1]. The largest burden occurs in low-and-middle income countries [2]. Childhood blindness is a significant contributor to the global economic burden of blindness and disability-adjusted life years. Control of blindness in children is closely linked to child survival [3].

The prevalence of childhood blindness in Ethiopia is 0.1%, accounting for over 6% of the total blindness burden [4]. Ocular surface disease, trauma, refractive error, and corneal scarring from vitamin A deficiency and measles are leading causes of pediatric ocular morbidity [5–7]. In Ethiopia, childhood blindness is avoidable in 89% of cases [8]. Prevention of childhood blindness, however, is challenging, as Sub-Saharan Africa has the lowest number of ophthalmologists per million population (2.7) worldwide [9]. As of 2017, there were only 3 tertiary pediatric eye centres for over 90 million population in Ethiopia [7]. Currently, there are no established training programs in pediatric ophthalmology in the country. To meet this unmet burden of childhood ocular disease, Ethiopian educational capacity to locally train highly qualified pediatric subspecialists must be sustainably enhanced. This may be achieved by following guidelines of the International Agency for the Prevention of Blindness and the World Health Organization Vision 2020: The Right to Sight initiative [10].

One existing model for the delivery of postgraduate medical training (i.e. residency and fellowship) in Ethiopia is the Toronto Addis Ababa Academic Collaboration (TAAAC) [11–15]. It was established in 2008 as a collaboration between the psychiatry departments of the University of Toronto (UofT) and Addis Ababa University (AAU), with the goal of developing a residency program to support the training and retention of psychiatrists in Ethiopia [16]. Over the past decade, the collaboration has built sustainable capacity in over 20 Ethiopian graduate programs, including family medicine, critical care, and emergency medicine [13, 17]. As of 2017, there were 222 graduates and over 90% remain in Ethiopia as faculty [13]. The TAAAC model strengthens graduate training capacity through 3-steps of global health collaboration, whereby a training program is first developed, graduates contribute to curriculum delivery as faculty, and programs are thereby sustained and undergo periodic review [16]. These programs are led by AAU faculty with support from UofT faculty, who periodically visit Ethiopia to deliver supplementary training.

AAU has a well-established ophthalmology residency program, making it an ideal location for the development of a subspecialty program in pediatric ophthalmology. Our aim was to assess the feasibility of implementing a pediatric ophthalmology and adult strabismus fellowship program at AAU following the TAAAC model.

## Methods

### Context and setting

In addition to being faculty of TAAAC-affiliated academic institutions, the senior authors were connected, as the current AAU Head of Ophthalmology (author S.T.S.) was a former Pediatric Ophthalmology Fellow trained at UofT. Prior to the launch of this situational analysis, several meetings, initiated by AAU faculty, were held between leaders of TAAAC, AAU and UofT Departments of Ophthalmology and their affiliated teaching hospitals (Menelik II Hospital, Addis Ababa and The Hospital for Sick Children, Toronto), to discuss the potential for a pediatric ophthalmology fellowship modeled by successful postgraduate medical education within TAAAC.

The AAU Department of Ophthalmology desired that the fellowship be modeled after the International Council of Ophthalmology (ICO) pediatric ophthalmology and strabismus subspecialty curriculum, following their successful implementation of the ICO residency curriculum [18]. The ICO curriculum offers international consensus on pertinent areas to be covered and recommended resources for fellowship training in pediatric ophthalmology and strabismus (including both pediatric and adult).

The ophthalmology residency program at AAU is a 4 year publicly funded program where residents are assigned by the Federal Ministry of Health through a matching program. Similarly, the salary of fellows will be covered by the government, but their selection will be done by the department.

### Data collection and analysis

We conducted a situational analysis, including a needs assessment, at Menelik II Hospital over a one-week period in February 2018. Availability of staff and resources (e.g. equipment, infrastructure) were quantitatively and qualitatively assessed through field observation in the eye clinics and operating rooms, and one-on-one discussions with AAU faculty, residents and administrators. Pediatric patient volumes were determined by retrospective chart review from the previous 3 years.

Local training needs were determined through informal feedback from the AAU residents and faculty on lectures delivered by UofT faculty, direct observation of resident clinical teaching and resident case log review. Team members interacted with local partners, including the Himalayan Cataract Project (HCP) and ORBIS International, to explore avenues of support for a future fellowship program. Meetings were held with TAAAC members to discuss the experiences of other successful academic partnerships.

### Development of fellowship curriculum

At the end of the week, key stakeholders held a strategic working meeting to discuss findings of the situational analysis and initiate curriculum development. Program feasibility was assessed through comparison of staff and resource availability to ICO recommendations. A program specification document was prepared based on the AAU Center for Academic Standards and Quality Enhancement (CASQE) guidelines.

## Results

### Staff expertise

The AAU Department of Ophthalmology faculty consisted of 11 fellowship-trained ophthalmologists, including 2 pediatric ophthalmologists (Table 1). At the time of assessment, two graduates of the AAU residency program were completing pediatric fellowships abroad and planned to return to AAU. Most subspecialists performed some pediatric surgical procedures, with follow-up care provided collaboratively. There was evidence of adequate faculty to support both educational and administrative fellowship components (Table 1).

**Table 1:** Department of Ophthalmology Staff at Addis Ababa University

Staff	No.
**Ophthalmologists**	11
Vitreoretinal Surgery	3
Pediatric Ophthalmology & Strabismus	2
Cornea and External Disease	2
Glaucoma	2
Oculoplastics	2
**Other (Pediatric Ophthalmology)**	
Operating Room Nurses	17
Clinic Nurses	5
Clinic Coordinators	2
Optometrists	2
Ocularists	1
Ophthalmic Assistants	1
Departmental Administrative Assistants	1
Ophthalmic Imaging Specialists	0
Opticians	0
Orthoptists	0

### Equipment and resources

Two examination rooms, adequately equipped with vision charts for different ages, slit lamps, tonometers, and other pertinent equipment, were utilized for pediatric ophthalmology clinics at Menelik II Hospital (Additional file 1).

Although adequate, additional space and equipment may be required as the fellowship program expands, such as a portable slit lamp and an indirect ophthalmoscope. At the time of assessment, there was capacity to expand to three rooms, which would support the fellow, faculty and resident. A larger eye hospital was also under construction within the Menelik II Hospital complex. Despite sufficient quantities of clinical equipment, repairs were often required, highlighting the need for robust standard operating procedures for resource maintenance and medical engineers formally trained in ophthalmic equipment.

Although not available on-site, most imaging modalities and visual field tests were available for pediatric usage in the private sector or at the neighbouring Black Lion Hospital. Four operating rooms were available for pediatrics at Menelik II, and one operating microscope had a teaching scope. Consumables, such as intraocular lenses and other non-reusable surgical materials, were funded through the national health insurance scheme, although availability was inconsistent. However, surgical volumes and utilization of consumables would not be expected to increase with the fellow operating under direct faculty supervision. There was sufficient access to inpatient facilities (40 beds), medical libraries and electronic medical journals.

### Pediatric ophthalmology and adult strabismus patient volumes

**Fig. 1:**
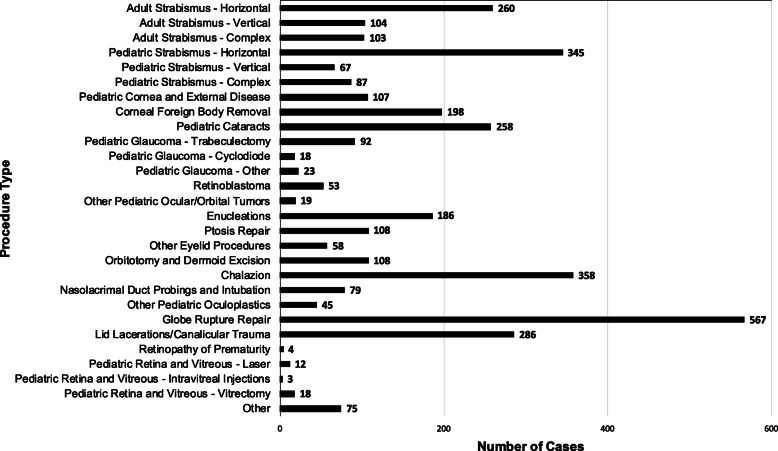
Total pediatric ophthalmology and adult strabismus surgical cases performed at Menelik II Hospital in 2017. The total number of cases (horizontal axis) performed over a 1-year period at a single academic teaching hospital in Addis Ababa, Ethiopia, distributed by procedure type (vertical axis)

Pediatric ophthalmology and adult strabismus cases seen at Menelik II Hospital totalled 14,627 medical and 3641 surgical in 2017, 13,488 medical and 3423 surgical in 2016, and 12,915 medical and 3197 surgical in 2015. The surgical procedures performed in 2017, including pediatric ophthalmology, pediatric and adult strabismus, are highlighted in Fig. 1. These reflect more than adequate volumes to support both the ICO guidelines (minimum 60 procedures as primary surgeon, at least 10 non-strabismus and at least 30 strabismus) and fellow participation in pre- and post-operative care.

### Residency program educational experience

The 4-year AAU ophthalmology residency program consisted of a structured teaching curriculum, weekly grand rounds, journal clubs, two required research projects, annual examinations and formal evaluations. At the time of assessment, there were 35 residents who rotated in pediatrics for 6 to 8 months. There were no established ophthalmology fellowship programs (since our visit, a glaucoma fellowship has been established). The presence of a well-established residency program would enable the pediatric ophthalmology fellow to become involved in clinical teaching, journal clubs and grand rounds.

Over the course of their training, AAU residents saw the following cases in children: approximately 480 cases of amblyopia, 336 cataracts, 120 glaucoma, 960 cornea and external disease, 96 retinoblastoma, 1320 ocular trauma, 526 oculoplastics, 1080 strabismus, 30 retina and 8 retinopathy of prematurity. They performed approximately 25 adult horizontal muscle strabismus procedures as primary surgeon and observed approximately 50 horizontal and 20 vertical pediatric strabismus procedures.

### Curriculum development and program specification

**Fig. 2:**
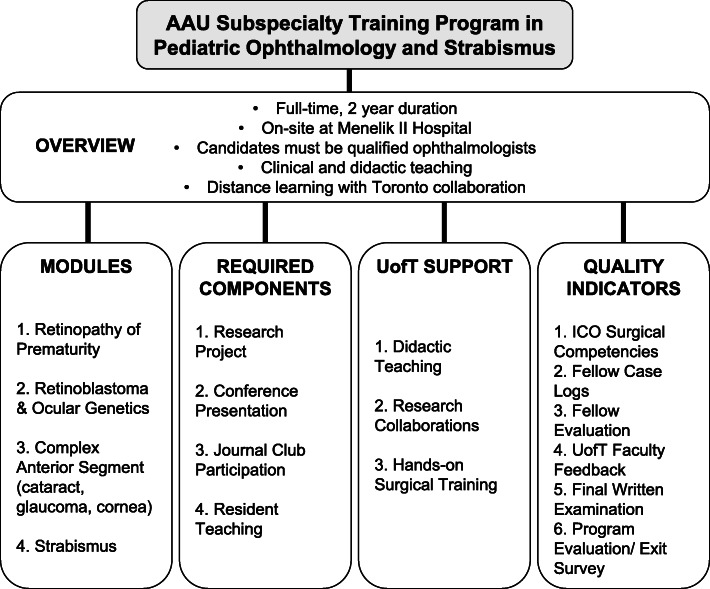
Components of the Pediatric Ophthalmology Fellowship Program. Schematic presentation of the components of the proposed fellowship program, including an overview, modules, requirements, pertinent areas identified for support from University of Toronto faculty, and quality standard indicators

There was sufficient staff expertise (4 anticipated full-time pediatric AAU faculty), equipment and patient volumes to meet the minimum ICO requirements for curriculum delivery, while maintaining the quality of resident education. Figure 2 summarizes the components of the proposed pediatric ophthalmology fellowship program.

Team members agreed on a full-time fellowship program on-site at Menelik II Hospital, with intended learning outcomes based on the ICO competencies and modified based on local needs. The fellowship would follow the ICO Ethical Guidelines for Ophthalmologists [19]. While the ICO suggests a minimum 12 continuous months of training, it was felt that a 2-year program would enhance training and allow the concurrent training of two fellows.

The resultant curriculum (initiated in this meeting and subsequently finalized) incorporates clinical, formal and informal didactic teaching. A policy document defines suitable surgical cases for fellows and residents, with fellows given priority for complex strabismus and pediatric intraocular procedures. Key modules identified by AAU faculty included retinopathy of prematurity (ROP), retinoblastoma, complex anterior segment and strabismus. UofT faculty will provide supplemental training for all subspecialties, with particular emphasis on ROP and retinoblastoma, for which there is most urgent need in Ethiopia.

The AAU faculty identified structured didactic teaching, research collaboration and hands-on surgical supervision as important roles for UofT faculty support. Although TAAAC programs traditionally involve 3 one-month UofT faculty visits annually [16], UofT faculty time constraints involving clinical, research, administrative and other educational responsibilities would be a potential challenge to implementing this model. A decision was made to limit the faculty visits to 2-weeks of concentrated surgical supervision, and to supplement training before and after on-site visits with remote didactic online instruction and telemedicine for discussion of select clinical cases.

Graduation requirements include completion of a formal research project and final written examination. Other quality standard indicators include review of surgical case logs, journal club and teaching contributions, publications and meeting presentations, faculty evaluations modelled on TAAAC formative assessments, and program evaluation by the fellow. The ICO Ophthalmology Surgical Competency Assessment Rubric (OSCAR) will be used [20].

The fellowship proposal requires internal and external assessments, and approval from the AAU Department of Ophthalmology, the College Academic Commission, Graduate Program office, and AAU Senate, which are currently in process.

## Discussion

Needs assessments are an effective means of identifying priorities for educational programs and international academic partnerships, in collaboration with local stakeholders [11, 15, 17, 21–28]. Observation of clinical cases enabled real world experience on how team members from both centres may deliver a collaborative training program.

TAAAC developed following the Ethiopian government’s “2008 University Capacity Building Programme” targeting university expansion, which led to demand for additional teaching faculty [13]. It is sustained by investment of both partner institutions, with AAU financially supporting UofT visits, and UofT contributing human resources [13]. This situational analysis identified sufficient staff expertise, equipment and patient volumes to facilitate a pediatric ophthalmology fellowship modeled by successful postgraduate medical education within TAAAC. AAU is an ideal location - the residency program has graduated over 120 ophthalmologists since its establishment in 1980, who now provide care throughout Ethiopia. It is anticipated that graduating fellows will provide care in both urban and rural communities, as those that receive fellowship sponsorship from other Ethiopian universities will return to those institutions [13]. The resultant pediatric ophthalmology workforce will inform future iterations of the Ethiopian Universal Eye Health Plan [29].

The traditional TAAAC model for UofT faculty visits was identified as a challenge for the planned pediatric ophthalmology program, due to competing faculty commitments. However, didactic teaching may be delivered online and telemedicine may facilitate clinical case discussion, thus supplementing the 2-week faculty visits. The ROP module is particularly suitable, as lectures may be conducted in real-time and retinal imaging discussed remotely. An ROP tele-education system was previously found to improve ROP diagnostic accuracy by ophthalmology residents [30]. Videoconferencing was also integrated into the AAU emergency medicine residency to supplement TAAAC faculty visits, although internet reliability was limited at that time [15]. Human capacity building in international telemedicine has been successfully modelled in other areas of Sub-Saharan Africa [31]. Distance education is becoming increasingly feasible as Ethiopian internet capacity expands. To address limitations in telecommunication capacity, AAU and UofT faculty applied for, and were awarded, a grant from the XOVA/Novartis Excellence in Ophthalmology & Vision Award. This has allowed the procurement of software and hardware to facilitate videoconferencing, capture of fundus images, and telemedicine consultation. A mixed online-onsite training format is currently being pilot tested at AAU, the results of which will inform the development of the fellowship program and form the basis of a future publication.

Ophthalmic equipment and infrastructure were adequate to support existing faculty and trainees, though needs are anticipated to increase with the addition of fellows performing independent examinations. As some patients need to be seen in the private sector for further diagnostic or therapeutic services, fellows will be trained at these centers through a private public partnership arrangement, with the goal of bringing these technologies and techniques to the public sector. While AAU has existing partnerships with NGOs such as Himalayan Cataract Project and Orbis International outside of the fellowship framework, there is potential for future collaboration to train a medical engineer to provide long-term support for the ophthalmology department. Such partnerships have been instrumental in supporting the training of pediatric nurses and an ocularist at Menelik II Hospital. Investment in equipment maintenance policies, inventories, standard operating procedures and onsite medical engineers will be even more critical following the construction of a larger eye center at Menelik II Hospital [32]. There may also be potential for collaboration with the UofT Institute of Biomedical Engineering through TAAAC.

ROP and retinoblastoma were identified as important fellowship modules. While pediatric patient volumes were adequate overall, ROP volumes were comparatively low. This may reflect the lack of a formal ROP screening program in Ethiopia, the distribution of 30 neonatal intensive care units in Addis Ababa, and the few pediatric ophthalmologists available for screening. A recent International Pediatric Ophthalmology and Strabismus Council survey found that the majority of countries lacking ROP screening strategies were in Africa [33]. Development of a national Ethiopian screening strategy is imperative as quality of neonatal care and infant mortality rates improve and ROP incidence increases. Similarly, there is growing importance to develop a national retinoblastoma strategy, modelled on other successful initiatives in Africa. Following its establishment in 2008, the multidisciplinary Kenyan National Retinoblastoma Strategy evolved to include consensus guidelines for care, a national pathology service and other capacity building initiatives [34]. Similar to the Ethiopian Universal Eye Health Plan, this strategic plan is endorsed by the national Ministry of Health to direct education and resources, and thereby improve health outcomes in these high-needs areas.

Ocular and periocular trauma account for nearly 25% of the pediatric surgical cases seen at Menelik II Hospital. While these patient volumes exceed minimum requirements for curriculum delivery, this highlights an important area for preventative eye care, in addition to other etiologies of childhood blindness such as trachoma and congenital glaucoma. Preventative eye care will serve as a major fellowship component, through both clinical, research and outreach initiatives.

The development of Ethiopian subspecialty training programs is critical, as a primary consideration for migration of African ophthalmologists is foreign educational opportunities [35]. Several eye care capacity building initiatives have expanded the ophthalmology workforce in Africa, including an academic partnership between Malawi’s College of Medicine and the University Eye Hospital Tübingen, Germany [36]. Residency curriculum, clinical guideline development and continuing medical education have been enhanced by a VISION 2020 LINK between the College of Ophthalmology of Eastern, Central and Southern Africa and the UK Royal College of Ophthalmologists [37]. The proposed fellowship aims to further expand the field of pediatric ophthalmology in Africa through sustained educational contributions.

Academic partnerships improve training in both contributing nations. Low-and-middle income countries benefit from research support and shared educational resources focused on addressing local needs. Benefits of collaboration for high-income countries include the formation of meaningful, long-term friendships [37], improved cultural competencies, understanding of disease in local contexts [36], and challenges of care accessibility, which may enrich care in the home nation [38].

## Conclusions

This situational analysis provided a way forward for the development of a pediatric ophthalmology fellowship, which would be the first of its kind in Eastern Africa. The on-site needs assessment at Menelik II Hospital demonstrated the feasibility of learning outcomes given a high volume of complex cases, qualified staff supervision and sufficient resources, and set a context for the academic partnership. The development of an academic partnership between Canada and Ethiopia is an exciting avenue for future collaborative initiatives, which may strengthen cultural competencies and provide benefits to both nations.

## Supplementary Information

**Additional file 1.** Availability of Equipment and Resources at Menelik II Hospital.

## Data Availability

All data generated or analysed during this study are included in this published article and its supplementary information files.

## Abbreviations

AAU: Addis Ababa University
UofT: University of Toronto
TAAAC: Toronto Addis Ababa Academic Collaboration
ICO: International Council of Ophthalmology
HCP: Himalayan Cataract Project
CASQE: Center for Academic Standards and Quality Enhancement
ROP: Retinopathy of prematurity
OSCAR: Ophthalmology Surgical Competency Assessment Rubric
