# Supplementary material for: Development of a pediatric ophthalmology academic partnership between Canada and Ethiopia: a situational analysis
Source: BMC Med Educ. 2020 Nov 16;20:438. doi: 10.1186/s12909-020-02368-y (PMC7670694; doi:10.1186/s12909-020-02368-y)
Supplement: Supplementary file 1 — Additional file 1. Availability of Equipment and Resources at Menelik II Hospital. [file 12909_2020_2368_MOESM1_ESM.docx]

| **Additional File 1. Availability of Equipment and Resources at Menelik II Hospital** | | |  |  |  |
| --- | --- | --- | --- | --- | --- |
|  |  |  |  |  |  |
|  | | **YES/NO** |  |  |  |
| **EQUIPMENT** | | |  |  |  |
| **Vision Charts** | |  |  |  |  |
| Near Fixation Targets and Vision Cards | | Y |  |  |  |
| Preferential Looking Tests | | N |  |  |  |
| Allen, Tumbling E or Landolt C | | Y |  |  |  |
| Lea or HOTV | | Y |  |  |  |
| Sloan, Snellen or ETDRS | | Y |  |  |  |
| **Imaging Modalities** | |  |  |  |  |
| A/B Scan Ultrasound | | N* |  |  |  |
| Ultrasound Biomicroscopy | | N* |  |  |  |
| External Photography | | N* |  |  |  |
| Standard Retinal Imaging | | N* |  |  |  |
| Wide-field Retinal Imaging | | N |  |  |  |
| Optical Coherence Tomography | | N* |  |  |  |
| Fluorescein Angiography | | N* |  |  |  |
| Corneal Topography | | Y |  |  |  |
| **Visual Field Testing** | |  |  |  |  |
| Goldmann | | N* |  |  |  |
| Humphrey | | N* |  |  |  |
| Frequency Doubling Technology | | Y |  |  |  |
| **Other Clinic Equipment** | |  |  |  |  |
| Tonometers (Tonopen, iCare, Goldmann,  Schiotz, Perkins) | | Y |  |  |  |
| Slit Lamp Biomicroscope | | Y |  |  |  |
| Portable Slit Lamp | | Y |  |  |  |
| Direct Ophthalmoscope | | Y |  |  |  |
| Indirect Ophthalmoscope | | Y |  |  |  |
| Condensing Lenses | | Y |  |  |  |
| Lensometer | | Y |  |  |  |
| Phoropter | | Y |  |  |  |
| Muscle Light | | N |  |  |  |
| Free Refraction Lenses | | Y |  |  |  |
| Pachymeter | | Y |  |  |  |
| Synoptophore | | Y |  |  |  |
| Retinoscope | | Y |  |  |  |
| Sensory tests | | Y |  |  |  |
| Prisms | | Y |  |  |  |
| Autorefractor | | Y |  |  |  |
| **Surgical Equipment** | |  |  |  |  |
| Operating Microscopes | | Y |  |  |  |
| Cryotherapy | | Y |  |  |  |
| Lasers (endolaser, 532 nm, selective laser  trabeculoplasty, 810 nm, indirect) | | Y |  |  |  |
| Intraocular lenses, implants and prosthetic eyes | | Y |  |  |  |
| **RESOURCES AND INFRASTRUCTURE** | | |  |  |  |
| **International Council of Ophthalmology Recommended** | |  |  |  |  |
| Pediatric Ophthalmology Clinic Exam Rooms | | Y |  |  |  |
| Inpatient Beds | | Y |  |  |  |
| Library and Electronic Resources | | Y |  |  |  |
| Radiology - Computed Tomography | | Y |  |  |  |
| Radiology - Magnetic Resonance Imaging | | N* |  |  |  |
| General Pediatrics Service | | Y |  |  |  |
| **Other** | |  |  | | |
| Operating Rooms for Pediatric  Ophthalmology | | Y |  |  |  |
| Administrative Facilities | | Y |  |  |  |
| Genetic Testing and Counselling | | N |  |  |  |
| Low Vision Rehabilitation | | N |  |  |  |
| Electrophysiology | | N |  |  |  |
| Subspecialty Pediatrics Services | | N* |  |  |  |
| Wet Lab | | Y |  |  |  |
| Research Ethics Board | | Y |  |  |  |
| Eye Bank of Ethiopia | | Y |  |  |  |
|  |  |  |  |  |  |
| **Abbreviations. *N* indicates resource is located offsite in private sector or neighbouring hospital*** | | |  |  |  |
